# Indirect Quantification of Glyphosate by SERS Using an Incubation Process With Hemin as the Reporter Molecule: A Contribution to Signal Amplification Mechanism

**DOI:** 10.3389/fchem.2020.612076

**Published:** 2020-12-18

**Authors:** Karen A. López-Castaños, Luis A. Ortiz-Frade, Erika Méndez, Enrique Quiroga-González, Miguel A. González-Fuentes, Alia Méndez-Albores

**Affiliations:** ^1^Centro de Química-ICUAP, Benemérita Universidad Autónoma de Puebla, Puebla, Mexico; ^2^Centro de Investigación y Desarrollo Tecnológico en Electroquímica (CIDETEQ), Pedro Escobedo, Mexico; ^3^Facultad de Ciencias Químicas, Benemérita Universidad Autónoma de Puebla, Puebla, Mexico; ^4^Institute of Physics, Benemérita Universidad Autónoma de Puebla, Puebla, Mexico

**Keywords:** surface enhanced Raman spectroscopy, quantification, glyphosate, reporter molecule, hemin

## Abstract

The indirect determination of the most used herbicide worldwide, glyphosate, was achieved by the SERS technique using hemin chloride as the reporter molecule. An incubation process between hemin and glyphosate solutions was required to obtain a reproducible Raman signal on SERS substrates consisting of silicon decorated with Ag nanoparticles (Si-AgNPs). At 780 nm of excitation wavelength, SERS spectra from hemin solutions do not show extra bands in the presence of glyphosate. However, the hemin bands increase in intensity as a function of glyphosate concentration. This allows the quantification of the herbicide using as marker band the signal associated with the ring breathing mode of pyridine at 745 cm^−1^. The linear range was from 1 × 10^−10^ to 1 × 10^−5^ M and the limit of detection (LOD) was 9.59 × 10^−12^ M. This methodology was successfully applied to the quantification of the herbicide in honey. From Raman experiments with and without silver nanoparticles, it was possible to state that the hemin is the species responsible for the absorption in the absence or the presence of the herbicide via vinyl groups. Likewise, when the glyphosate concentration increases, a subtle increase occurs in the planar orientation of the vinyl group at position 2 in the porphyrin ring of hemin over the silver surface, favoring the reduction of the molecule. The total Raman signal of the hemin-glyphosate incubated solutions includes a maximized electromagnetic contribution by the use of the appropriate laser excitation, and chemical contributions related to charge transfer between silver and hemin, and from resonance properties of Raman scattering of hemin. Incubation of the reporter molecule with the analyte before the conjugation with the SERS substrate has not been explored before and could be extrapolated to other reporter-analyte systems that depend on a binding equilibrium process.

## Introduction

N-phosphonomethyl glycine, known as glyphosate (GLY), is the main active ingredient of one of the most used herbicides worldwide (Avino et al., 2020). GLY presents a non-selective systemic mode of action; once it is absorbed by plants mainly through the foliage, the substance has the ability to translocate to metabolic sinks where it disables the Shikimate pathway of enzyme 5-enolpyruvylshikimate-3-phosphate synthase (EPSPS) and interrupts the synthesis of aromatic amino acids involved in the plant growth (Turner, 2020).

Since the 1970s, glyphosate has been used in an excessive and deregulate manner in agriculture. It is currently applied in field crops, including fruits, vegetables and cereals, for both conventional and genetically modified (GM). As a result, glyphosate residues are commonly found in environmental, biological, and food samples (Steinborn et al., 2016; Avino et al., 2020).

The glyphosate residue intake is a latent risk for human health since the herbicide has been cataloged as a potential carcinogen according to IARC, the International Agency for Research on Cancer from the World Health Organization (IARC, 2017). Despite the importance of glyphosate, its stability to hydrolysis and the lack of chromophore or fluorescent groups complicate its analytical determination, besides the difficulties of being separated in food matrices due to the similarity with natural amino acids.

At present, the most employed analytical technique to quantify glyphosate in food is liquid chromatography-tandem mass spectrometry (LC-MS/MS). However, this technique is expensive and requires sophisticated equipment and qualified personnel, in addition to the possibility of matrix effects (Avino et al., 2020). For this reason, research efforts are being focused on the development of alternative methods for analytical applications. In this context, Surface Enhanced Raman Spectroscopy (SERS) is among the most robust options to be employed as an alternative or pre-screen method before the use of a routine analytical technique in a wide variety of fields, including food safety (Zhao et al., 2018; Lin et al., 2020). However, this technique still presents serious drawbacks that have impeded its use as a routine analytical technique at the level of LC-MS/MS (Pérez-Jiménez et al., 2020). SERS is based on the amplification of the Raman response of an analyte when it is adsorbed on or in close proximity with the plasmon surface of metals such as Au, Ag, or Cu, and it is capable of single-molecule identification in some cases (Demirel et al., 2018). To maximize the SERS signals, it is mandatory to combine the optimal performance of the plasmonic substrates (electromagnetic contribution, EMM) with the chemical contribution (CHEMM) of the adsorbate and from the adsorbate-substrate interaction under the effect of the incident light (Pilot et al., 2019). Examples of chemical contributions are the charge transfer between the metal and the target molecule, or vice versa, and/or non-resonant effects (static charge transfer) by the adsorption process of the molecule in its electronic ground state (Cui et al., 2010; Pilot et al., 2019). Thus, the achieved sensitivity in SERS measurements will depend on the chemical properties of the analyte and on the optimization of the Raman signal by EMM and CHEMM. So far, optimizing the SERS signal is commonly addressed through the SERS-active metal substrates (EMM). Despite the great advances in this area, the relatively high cost, low homogeneity and reproducibility of the substrates result in some of the most important drawbacks for practical applications of SERS (Mosier-Boss, 2017).

Detection of analytes by SERS has been conducted in both direct and indirect forms. The indirect form (IF) is for analytes that are not able to be adsorbed or to be close to the metal surface, resulting ideal when the target molecule possess low or null Raman vibration modes, or when the selectivity needs to be enhanced, such as in the case of oligonucleotide sequences distinction (Pyrak et al., 2019) or biomarker detection (Li et al., 2017). The IF correlates the SERS spectrum changes of a metabolite, a reaction product, or a Raman reporter molecule (RM), attached on the surface of the SERS substrate, with the concentration of the target analyte (Xu et al., 2018; Pilot et al., 2019). The use of reporter molecules is the most common way to address the indirect detection, especially in biological samples where the combination of RMs with specific antibodies also adsorbed on SERS nanoparticles (SERS tag) forms part of the detection strategy. RMs usually are small in size, present high Raman cross-sections at the selected wavelength, are photochemically stable, and show a preference for the plasmonic metal employed. They also present phenomena that contribute to the enhancement of the Raman signal, such as Raman resonance scattering properties that may result in contributions of chemical nature to the SERS signal (Li et al., 2018). Despite the wide variety of RMs reported in the literature and even of their commercial availability, the scope of their application is still underdeveloped (Sánchez-Purrà et al., 2018).

Reported methodologies to detect and quantify GLY by SERS include the direct and the indirect measurement ways. As indirect detection, we can mention de following works: Torul et al. (2010) reported an indirect detection that includes the use of gold nanorods (AuNR) derivatized with 4-mercaptophenylboronic acid as the reporter molecule. These particles were mixed with GLY in methanol, left for interaction during a specific time, and then deposited onto a silicon wafer by evaporation of the solvent for SERS measurements. Attomolar detection levels (1 × 10^−16^ mM) were achieved under this strategy, and the sensor was tested in tomato juice. Tan et al. (2017) reported a SERS strategy for the quantification of organophosphate pesticides (OPPs), including glyphosate, by using osmium carbonyl clusters on the surface of gold nanoparticles as SERS probes in a liquid medium. The analytical strategy contemplates the inhibition of thiocholine (the acetylcholinesterase catalyzed hydrolysis product of acetylthiocholine) and the subsequent decrease of thiocholine-induced aggregation of the SERS probes when OPPs are present in the sample. Changes in their aggregation modify the CO stretching vibration signal of the SERS probes at the mid-IR region (1,800–2,200 cm^−1^), making the quantification of glyphosate possible. The limit of detection was 0.1 ppb (5.91 × 10^−10^ M), and the method was evaluated in spiked beer samples. Xu et al. (2018) proposed a method based on the SERS activity of silver nanoparticles (AgNPs) in a colloidal medium through the detection of purple color dye (PD), a product formed during the derivatization of GLY with ninhydrin. The reported limit of detection (LOD) was 1.43 × 10^−8^ M, and the methodology was evaluated in water samples. However, extrapolation of the methodology to foodstuff may be complicated by the well-known reaction of ninhydrin with amino acids. In the case of direct detection, De Góes et al. (2017) detected the herbicide by using Ag colloidal solutions. Detection was possible from the interaction of the negatively charged AgNPs with the respective species of GLY at different pH values (4–8). The LOD was 19 mM and showed applicability in the evaluation of tap water samples in the presence of interferents, such as glufosinate-ammonium salt, AMPA, sodium chloride, and with commercial glyphosate-based herbicides.

Hemin is the hemoglobin oxidation product; it is a protoporphyrin IX containing an iron (III) center (Heme B) with a chloride ligand. As a biosensor element, it has been used for electrochemical detection of 2,4,6 trichlorophenol (Zhang et al., 2018), ascorbic acid, dopamine, uric acid (Zou et al., 2015), and glucose (He et al., 2016). Likewise, the hemin/G-quadruplex Dnazyme, an artificial enzyme with peroxidase activity, has been employed to develop electrochemical and colorimetric detection of a variety of chemical and biological targets (Alizadeh et al., 2017). The use of hemin as a reporter molecule for the optical determination of analytes by Raman has not been explored; however, it presents multiple advantages such as a variety of electronic transitions in the visible and near*-*infrared regions and its well-characterized Raman spectra at different excitation wavelengths (Franzen et al., 2002). Therefore, in this work, hemin chloride was used as a Raman reporter molecule to determine glyphosate. Also, in order to potentiate the enhancement and stability of the Raman signal, the use of an incubation process between the Raman reporter and the analyte, previously to SERS measurements, was tested for the first time. The SERS experiments were conducted on Si-AgNPs substrates using the wet mode of spectrum acquisition developed in our research group (“the drop technique”), which allows the *in situ* evaluation of the adsorption process on a simple and easy way.

## Materials and Methods

### Chemicals and Materials

AgNO_3_ 99%, hydrofluoric acid (HF, 48%), sodium hydroxide (NaOH, 97%), sodium borate decahydrate (Na_2_B_4_O_7_·10H_2_O), hemin (C_34_H_32_ClFeN_4_O_4_, FW 651.96 g/mol), were purchased from Sigma-Aldrich. Glyphosate (99.5%) was procured from Chem Service. All reagents were used without further purification. Silicon wafers (Si) type P, doped with boron (B), orientation (100), and resistivity of 15–25 (Ω cm) were purchased from Siltronic AG. Deionized water with a resistivity of ~18 MΩ cm was obtained by using a Millipore MilliQ Plus water purification system. Acetone (C_3_H_6_O, 98%) was obtained from CTR Scientific, and nitrogen gas (99.9%) was purchased from Infra.

### Preparation and Characterization of Si-AgNPs Substrates

The Si-AgNPs substrates were synthesized by chemical deposition. Pieces of 1 cm^2^ of polished p-type (100) Si wafers with a resistivity range of 15–25 Ωcm (electronic grade) were used as substrates. Si pieces were subjected to exhaustive washing with water for 10 min and acetone for 5 min. After that, the pieces were rinsed with water. Then, Si pieces were immersed for 7 min into the deposition solution prepared with 0.2 mL of 48% HF and 10 ml of AgNO_3_ 5.88 × 10^−4^ M. HF reacts with Si producing soluble H_2_SiF_6_ and electrons. The electrons reduce the Ag^+^ ions of the plating solution, producing elemental Ag particles on the surface of Si. A thorough explanation of the reaction mechanism is given in Aca-López et al. (2020). After deposition, the Si pieces were immediately immersed in distilled water to stop the deposition process. To prevent oxidation of the deposits, every sample was dried with a flux of nitrogen.

For the optical characterization of the Si-AgNPs, UV-Vis diffuse reflectance measurements were performed using a Si substrate spectrum as a reference. The measurements were carried out using a Cary 50 UV-Vis spectrophotometer from Varian Instruments, equipped with an integrating sphere. A homemade sample holder made of Teflon was used to fix the samples during the measurements. UV-Vis diffuse reflectance spectra were corrected by subtracting the spectrum of the Teflon holder. All spectra were normalized to the maximum. 1-R spectra are reported for all samples.

The morphological characterization, AFM images were obtained using a SmartSPM^TM^ 1000 atomic force microscope (Horiba Scientific) with the “Top mode” tool. Silicon cantilevers (AppNano) of 52 μm width, 0.8–8.9 N m^−1^ spring constant, and 36–98 kHz resonance frequency in the air were used. Scan speed of 1.0 Hz and 450 × 450 pixels per line resolution were employed. Images were processed using the Gwydion 2.30 software.

### Sample Preparation

Solutions of hemin were prepared fresh by dissolving hemin chloride to a concentration of 25 μM in 0.25 M borax (pH 9.26). Glyphosate solutions (1 × 10^−4^, 1 × 10^−3^, 1 × 10^−2^, 0.1, 1 and 10 μM) were prepared by dilution of a 0.01 M glyphosate stock solution. The sample containing glyphosate (deionized water or spiked honey solutions) was incubated with a hemin–borate solution in a volume ratio of 1:1 and then left to rest for 48 h at −4°C without light exposition. UV-Vis studies of hemin and hemin-glyphosate solutions were carried out using a Cary 50 UV-Vis spectrophotometer from a Varian instrument. With the same technique, hemin concentration was corroborated by measuring the absorbance of the solution at λ = 385 nm (€ = 5.84 × 10^4^ cm^−1^ M^−1^).

### Preparation of Real Spiked Samples

A series of honey samples artificially contaminated with glyphosate at concentrations of 0.1 nM, 1 nM, 10 nM, 100 nM, 1 μM, and 10 μM were prepared as follows: 1 g of honey was weighed into a 5-mL volumetric flask and filled with a solution of 0.25 M borate. The sample was diluted with the necessary volume of borate solution to allow the honey dissolution; after that, 25 μM of a solution with the desired concentration of glyphosate was added to the mixture, and the volume completed to 5 mL with a borate solution. It was necessary that this solution was adjusted to pH 9.2 with 1 M NaOH due to the acidity of the honey.

### Raman Spectroscopy Measurements

Raman spectra were collected using a Micro-Raman system (Xplora Plus microscope from Horiba) equipped with a confocal microscope (Olympus BX51). Lasers of 532 and 780 nm wavelength excitation were employed at 20 and 100 mW power, respectively. The instrument was calibrated using the 520.71 cm^−1^ band of a silicon wafer. All spectra were obtained in aqueous media using the “drop technique.” A 10x objective was used. The acquisition parameters of the spectra were set to 1,200 g·mm^−1^ grating, slit 200 (μm), hole 300 (μm), 12 s of acquisition time, and an accumulation of five spectra. The control of the equipment for data acquisition and preliminary analysis was carried out with LabSpec6 software.

### The “Drop Technique”

**Figure 1:**
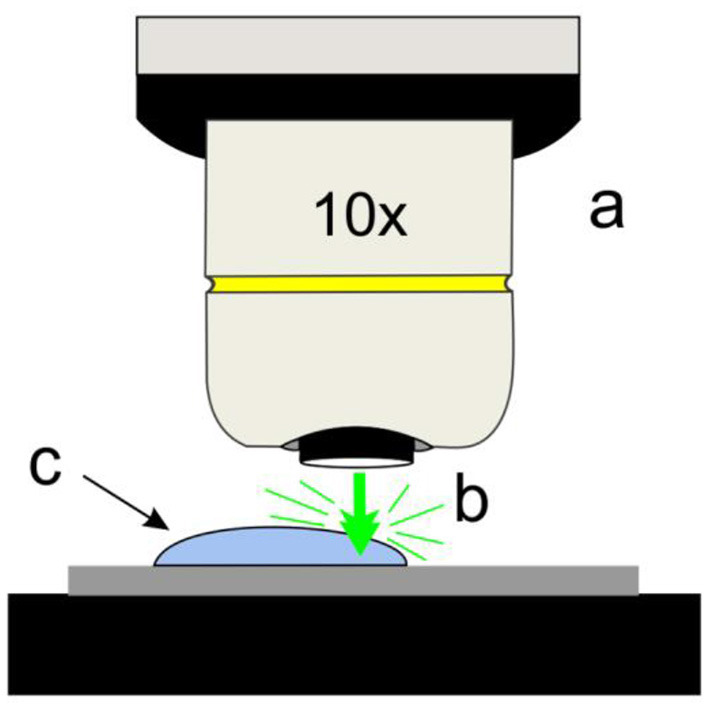
Simplified scheme of the *drop technique*: (a) objective, (b) laser beam, (c) drop of sample on Si-Ag NPs substrates.

SERS measurements were carried out using the “drop technique,” which consists of the following steps: (1) the Raman microscope is set to focalize the surface of the SERS substrate with the aid of a CCD camera; (2) a drop of the analyte is deposited on the surface of the SERS substrate by using a micropipette; (3) the measurement is performed on the droplet at 10 μm from its boundary. The acquisition parameters are selected in such a way to avoid the evaporation of the droplet during the measurement. The Raman experimental setup is depicted in Figure 1.

Through the use of this new drop technique, it is possible to carry out Raman experiments on SERS substrates with liquid media in a simple form, in comparison with other strategies reported in the literature, such as microfluidic system (LoC-SERS) (März et al., 2011) or on a drop of the liquid sample placed on the SERS substrate, which is covered with a thin coverslip (Peters et al., 2015).

## Results and Discussion

### Characterization of Si-AgNPs Substrates

**Figure 2:**
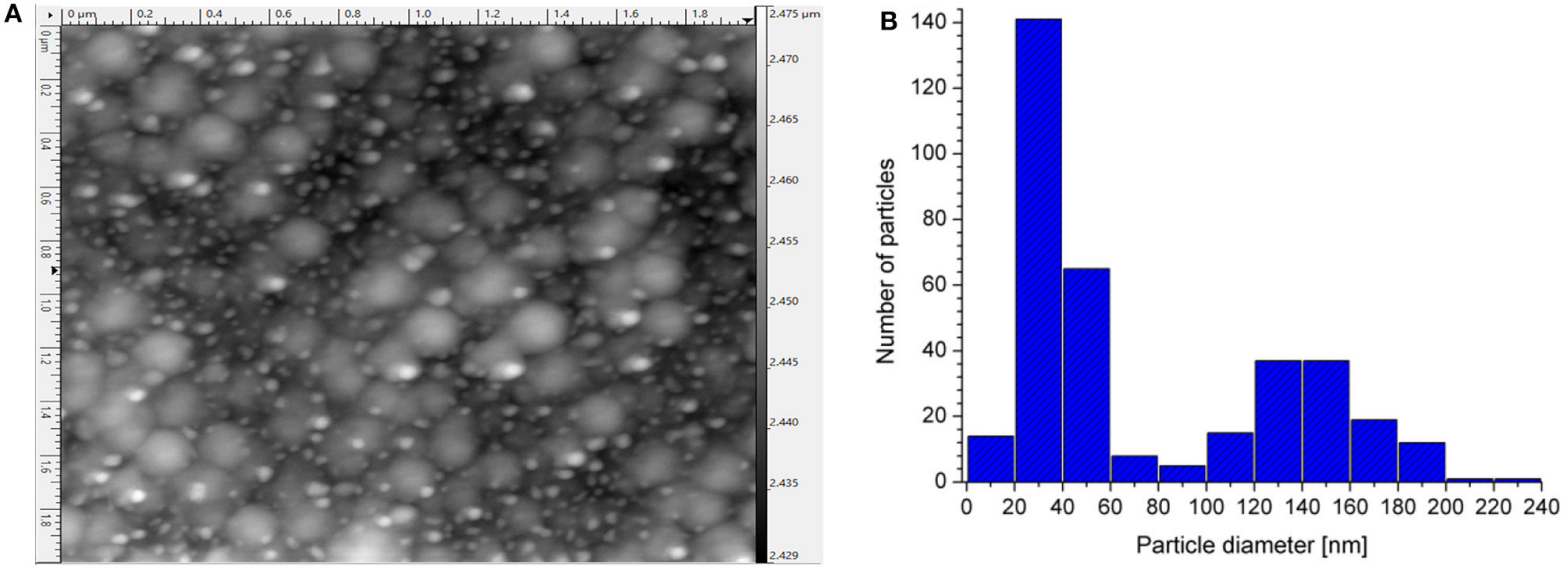
**(A)** Typical AFM micrograph of the Si-AgNPs substrates. **(B)** Histogram of the set of Ag nanoparticles from 2A micrograph.

The maximum electromagnetic contribution to the SERS intensity can be obtained from the Si-AgNPs substrates and corresponds to the surface plasmon resonance (LSPR) of the silver nanoparticles. AFM and diffuse reflectance UV-Vis spectroscopy were used in order to correlate the topographic characteristics of the silver deposit on silicon with the plasmonic response of the Si-AgNPs substrates. Figure 2A shows a typical AFM image of the Si-AgNPs substrate used for this study. It can be observed that Ag nanoparticles have a dispersion of sizes; however, most of the particles tend to be semi-spherical. The corresponding histogram of the size distribution of the Ag particles is presented in Figure 2B.

One can identify two main groups of particle sizes. One group has an average size of 140 nm, and about 60% of the particles of this group have sizes between 121 and 160 nm. The other group has sizes below 60 nm, where 90% of the particles have sizes between 21 and 60 nm. A mixture of particles of different sizes can be observed through the sample. In most of the reports of SERS substrates, the authors try to obtain monodisperse particles (Lee et al., 2019; Chen et al., 2020), but the techniques are more complicated than ours. The great advantage of the substrates of the present work is their simplicity and low fabrication cost.

Dispersion of size in plasmonic particles produces a broad plasmonic spectrum that could be useful for performing SERS at different wavelengths (Mao et al., 2020). A similar effect could be also obtained with dendrites (Lu et al., 2020) or particles with a dispersion of forms (Yin et al., 2020).

**Figure 3:**
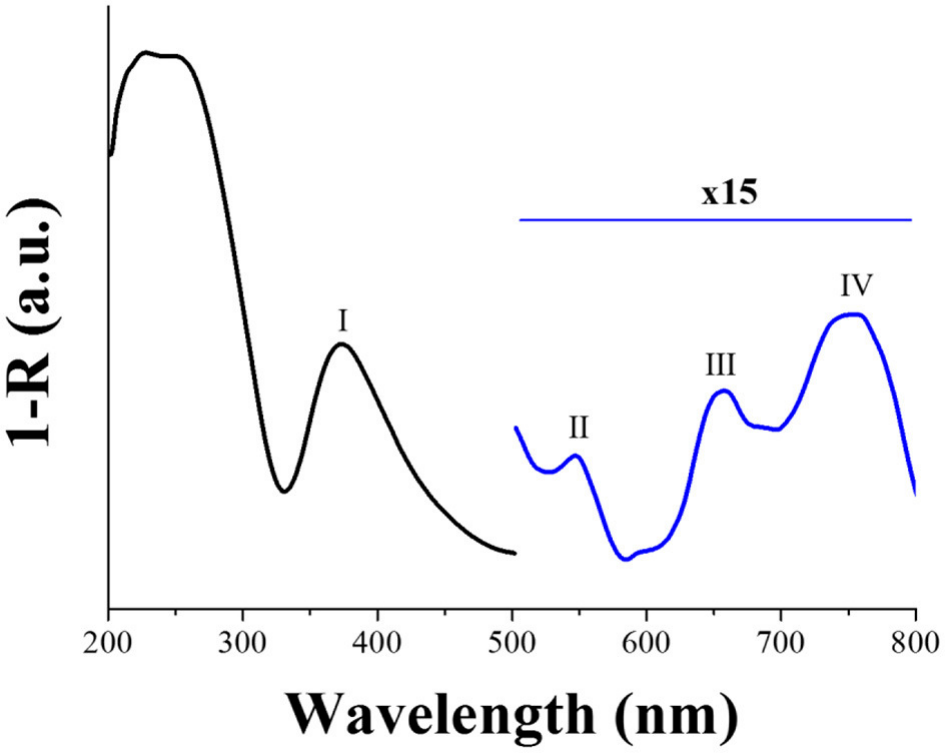
Graph of 1-R vs. wavelength. Absorption bands indicated as I, II, III, and IV, respectively.

The plasmonic response of the Si-AgNPs substrates was evaluated by diffuse reflectance UV-Vis spectroscopy. Figure 3 shows a graph of 1-R vs. λ. Four Localized Surface Plasmon Resonances (LSPRs) were identified: band I (371 nm), band II (543 nm), band III (657 nm), and band IV (752 nm). Similar multi-LSPRs were reported by Kosović et al. (2015) for silver nanoparticles exhibiting comparable morphological features to the Si-AgNPs substrates. The width of the most intense LSPR band (band I) is the result of the broad size distribution of particles, while the appearance of the rest of the bands (II, III, and IV) is most likely due to the different aspect ratios of the particles and their coalescence (Sharma et al., 2020).

Spherical particles exhibit one resonance band due to the presence of a single dipole; in the case of particles of 40 nm size, the band is intense and centered at about 400 nm (Lee et al., 2008). However, for quasi-spherical particles with an aspect ratio larger than 1, a double resonance occurs: the intense SPR band blue shift and an extra band appears at wavelengths above 500 nm (Amirjani and Haghshenas, 2018). On the other hand, an increase of the aspect ratio or the overall size of the particle provokes a red-shift of this additional peak (Sharma et al., 2020).

Thus, the origin of the bands at 371 nm and 543 nm in Figure 3 could be arising from semi-spherical particles with sizes below 60 nm. Likewise, bands at 371 and 657 nm could be originated from larger particles, as those with an average size of 140 nm, as reported by Kosović et al. (2015). Therefore, the band at 371 nm may result from the contribution of both particle sizes, which explains its broadness.

The band at 752 nm is due to the coalescence of the larger particles. As it can be observed in the micrograph (Figure 2A), groups of particles are found. It is well-known that the coalescence of particles generates multipole oscillations and new plasmonic modes at higher wavelengths (Amirjani and Haghshenas, 2018). Additionally, low-intensity shoulders were also observed at about 600 and 680 nm. These additional modes may also be related to the aforementioned Ag aggregated particles, specifically to their interaction. It is reported that a red-shift as large as 70 nm in the Raman peak position may occur if particles are close together (when the ratio of the gap over the particle diameter is smaller than 0.1). A similar shift occurs when contiguous particles differ in size (Drozdowicz-Tomsia and Goldys, 2011).

### UV-Vis Spectroscopy of Hemin-Glyphosate Incubated Solutions

It has been previously reported that the UV-Vis spectra of hemin dissolved in borate solution show multiple electronic transitions in the visible and near*-*infrared wavelength regions (Wood et al., 2004). However, the spectral behavior of aqueous solutions of hemin changes as a function of time (Maehly and Akeson, 1958). Considering the above information, in this work the incubation of a mixture of solutions of glyphosate (at different concentrations, from 1 × 10^−4^ to 10 μM) and hemin (25 μM) in a volume ratio of 1:1 was carried out for 48 h in order to allow the binding equilibrium to occur during the incubation exposition process and before SERS experiments.

**Figure 4:**
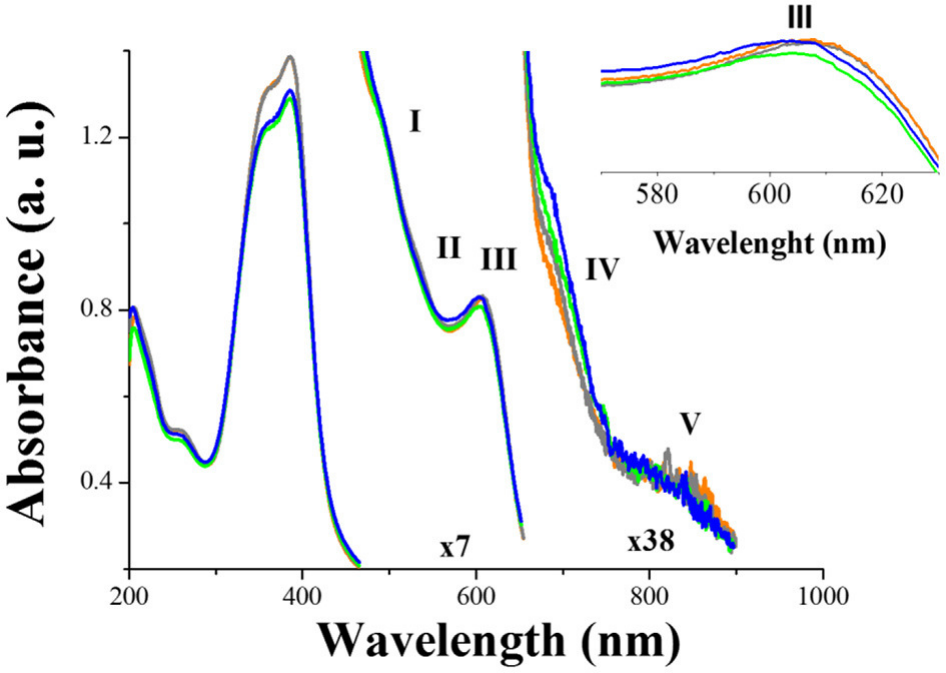
Absorbance spectra of: fresh solution of 25 μM hemin in 0.25 M borate solution (-); fresh mixture of hemin-borate and 1 μM glyphosate in borate (-); 25 μM of hemin dissolved in 0.25 M borate solution and after 48 h of incubation (-); mixture of hemin-borate and 1 μM glyphosate in borate after 48 h of incubation (-). The incubation was made at −4°C without light exposition. Inset: Amplification in the 550–630 nm range.

The characterization by UV-Vis spectroscopy of the hemin-glyphosate (1 μM) mixture before and after incubation is shown in Figure 4. For comparison, the absorption spectra of 25 μM hemin solution in borate, freshly prepared and 48 h aged are added.

All spectra present the same pattern as the freshly prepared hemin-borate solution: the characteristic partitioned Soret band B of the formation of hemin dimmers is present with a maximum intensity at 383 nm (Aratani et al., 2002). In the range from 450 to 900 nm, several bands are observed [I (493 nm), II (531 nm), III (610 nm), IV (695 nm), V (817 nm)]. Bands I and II, known as Q bands, are assigned to vibronic components of porphyrin ring π → π* transition modulated by the Fe(III) ion (Toader et al., 2013). Bands III, IV, and V are associated with charge transfer (CT) from dπ orbitals in the iron atom to the porphyrin ring with a variable contribution of the π-π* transition (Arbelo-López et al., 2018). However, bands IV and V are absent when hemin concentration is <15 μM (see Supplementary Figure 1) as it has been already reported (Nath et al., 2017). Thus, the presence of these bands indicates the molecular aggregation of hemin (Wood et al., 2004), and therefore the presence of excitonic interactions (Webster et al., 2009).

After aging, the spectral change of the hemin-glyphosate mixture and the hemin-borate solution is practically the same: The Soret band undergoes hypochromicity of about 3.5%, with no change in its adsorption position and bandwidth, which indicates the dissociation of hemin dimers over time. The Q bands do not show any modification; therefore, there is no change in the local environment of the hemin aggregates, even in the presence of glyphosate (Liu et al., 2018). Bands III and IV of hemin in the hemin-glyphosate spectra are slightly affected by the incubation process. Both bands undergo a slight hypochromic shift (blue) of about 3 nm (broken curves), indicating that the binding step for reaching equilibrium involves a subtle increase in the overlap between π porphyrin and metal dπ (dxz or d dxz) orbitals without an influence in the π to π* energy gap in porphyrin electronic spectra (Aarabi et al., 2019).

The analysis of UV spectra in Figure 4 does not show evidence of a strong interaction between glyphosate and hemin. However, since experiments were performed at a pH of 9.2, hemin exists predominantly in the form of dimmers, with the axial OH– ligands pointing outwards (Scolaro et al., 2002). Meanwhile, the dominant species of glyphosate is HL^2−^, with its two molecular ends negatively charged by the deprotonation of one oxygen at the carboxylate and phosphonate groups (Ehrl et al., 2018; Lopes Catão and López-Castillo, 2018). Therefore, glyphosate would be expected to interact with OH ions by hydrogen bonds.

### SERS Spectra of the Hemin-Glyphosate Mixture After the Incubation Process

When the laser used in SERS experiments corresponds to the excitation of LSPR of metal nanoparticles with a simultaneous electronic absorption of the molecule adsorbed on the substrate, an enhancement of the Raman signal additional to that corresponding to the electromagnetic contribution arises. This phenomenon is known as Resonance Raman Scattering (RRS) (Murgida and Hildebrandt, 2001). At 780 nm, the onset of the electronic transition assigned as band V is observed in hemin and the mixture of hemin-glyphosate solutions after incubation process (Figure 4): the signal is a charge transfer (CT) transition and involves promotions between π porphyrin and metal dπ (dxz or d dxz) orbitals (Wood et al., 2004). At this wavelength value, the Si-AgNPs substrates exhibit a LSRP signal (band IV) (Figure 3). Thus, the SERS response of both solutions (hemin and hemin-glyphosate) must be composed of contributions of the electromagnetic and electronic structure of chemical species due to the resonance enhancement effect.

**Figure 5:**
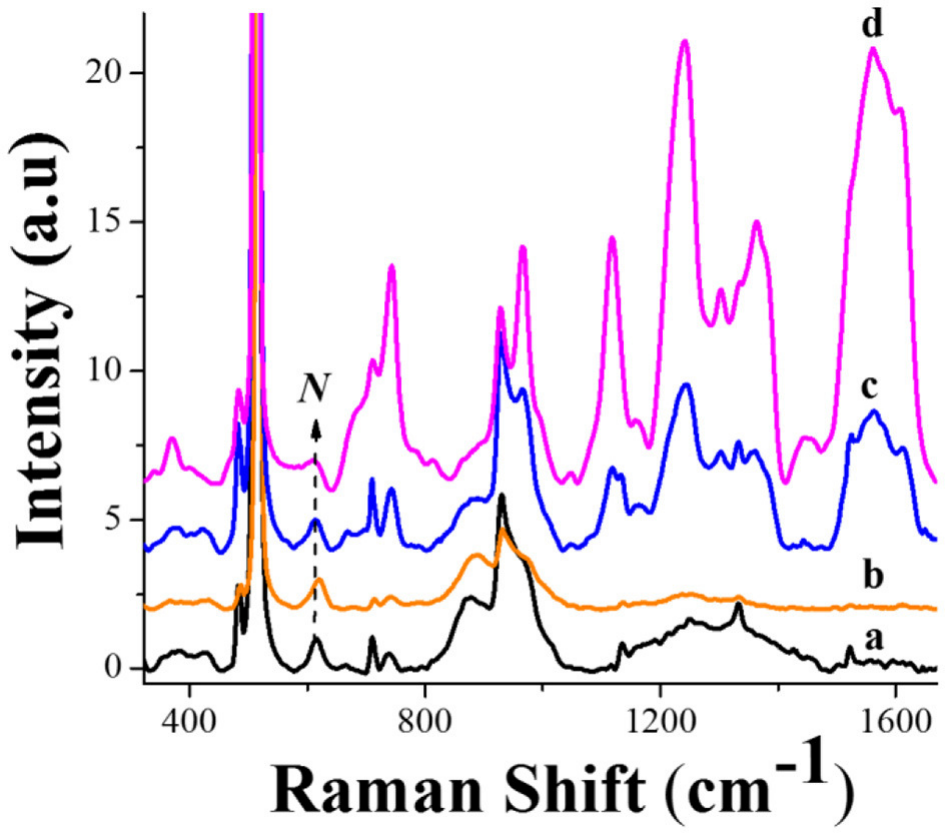
SERS spectra of the systems: 0.25 M borate solution (curve a), 1 μM GLY in 0.25 M borate solution (curve b), 25 μM of hemin in 0.25 M borate solution (curve c), and a mixture of the solutions of 25 μM of hemin in borate with 1 μM of glyphosate in water (curve d) after 48 h incubation.

Figure 5 shows the SERS spectra of the systems: 0.25 M borate solution (curve a), 1 μM GLY in 0.25 M borate solution (curve b), 25 μM of hemin in 0.25 M borate solution (curve c), and the hemin-glyphosate mixture containing 1 μM of glyphosate (curve d) after incubation. The most intense band at 515 cm^**–**1^ corresponds to the silicon wafer.

The SERS spectrum of borate solution (Figure 5, curve a) presents seven defined bands at 511, 613, 745, 930, 1,136, 1,333, and 1,524 cm^−1^. All signals except for the one at 745 cm^−1^ come from the silicon wafer (Supplementary Figure 2), while the band at 745 cm^−1^ appears in the presence of the borate medium and can be assigned to the vibrations of the tetrahydroxy borate ion ${\left(\text{B}(\text{OH}\right)}_{4}^{-}$) near the Si-AgNPs substrate (Yongquan et al., 2013). However, bands at 613 cm^−1^ and 930 cm^−1^ can also possess the contributions from B_3_O_3_${\left(\text{OH}\right)}_{4}^{-}$ ions and from the ${\text{BO}}_{3}^{3-}$ trigonal unit of the Na_2_B_4_O_7_ molecule (Norrel et al., 2003; Yongquan et al., 2013).

Curve b of Figure 5 corresponds to the borate-glyphosate system, and the spectrum presents the same behavior as in pure borate solution, with a decrease in the total Raman intensity. The lack of glyphosate signals is consistent with the behavior obtained by the water-glyphosate system (Supplementary Figure 2) at the same herbicide concentration. The low affinity of glyphosate for the Ag surface at the herbicide concentrations used in this work was reported recently by Feis et al. (2020).

The SERS spectrum of hemin-borate solutions after 48 h of their preparation (Figure 5, curve c), adds bands at 967, 1,121, 1,244, 1,303, 1,366, and 1,558 cm^−1^, and provokes the enhancement of the 930 and 745 cm^−1^ bands, suggesting the co-adsorption of ${\text{B}(\text{OH})}_{4}^{-}$ with hemin.

When glyphosate solution is incubated with the hemin solution (curve d), the SERS spectra do not show any new bands associated with the herbicide; however, the hemin peaks show an increase in intensity. Table 1 summarizes the assignments of the hemin bands where modes are designed considering the D_4h_ point group symmetry.

**Table 1:** Band assignments, symmetry term, and local coordinates from SERS spectra for hemin in borate after incubation during 48 h.

**Wavenumber (cm^**−1**^)**	**Assignment**	**Symmetry**	**Term local coordinate**
341	ν8	ν(Fe-N)	A1g
372	ν35	δ(CβCcCd)_6,7propionate_	A1g
407		δ(CβCaCb)_4−vinyl_	
426		δ(CβCaCb)_2−vinyl_	
741	ν15	ν (pyr breathing)non-totally sym	B_1g_
932	ν 46	δ (pyr deform)asym	E_u_
968	ν32+ν35	δ (porph def)+ δ (pyr transl)	A1g
1,120		ν (Cb-Cα (vinyl)+ δ(Cb-R) ^*^	
1,246	ν13	δ (C_m_H) non-totally sym	B1g
1,301		δ (CaH =)	
1,366	ν4	ν (Ca = N)sym	A1g
1,442		δ (= CbH2)sym	
1,565	ν2	ν (C_b_C_b_)sym	A1g
1,612		ν (Ca = C_b_)	

*Excitation wavelength: 780 nm*.

*ν = in-plane stretch*.

*δ = deformation mode*.

*Based on the works of Wood et al. (2011), Hu et al. (1996), and Wood et al. (2004)*.

* *Symmetry based from Desbois et al. (1984)*.

### Origin of the SERS Effect

**Figure 6:**
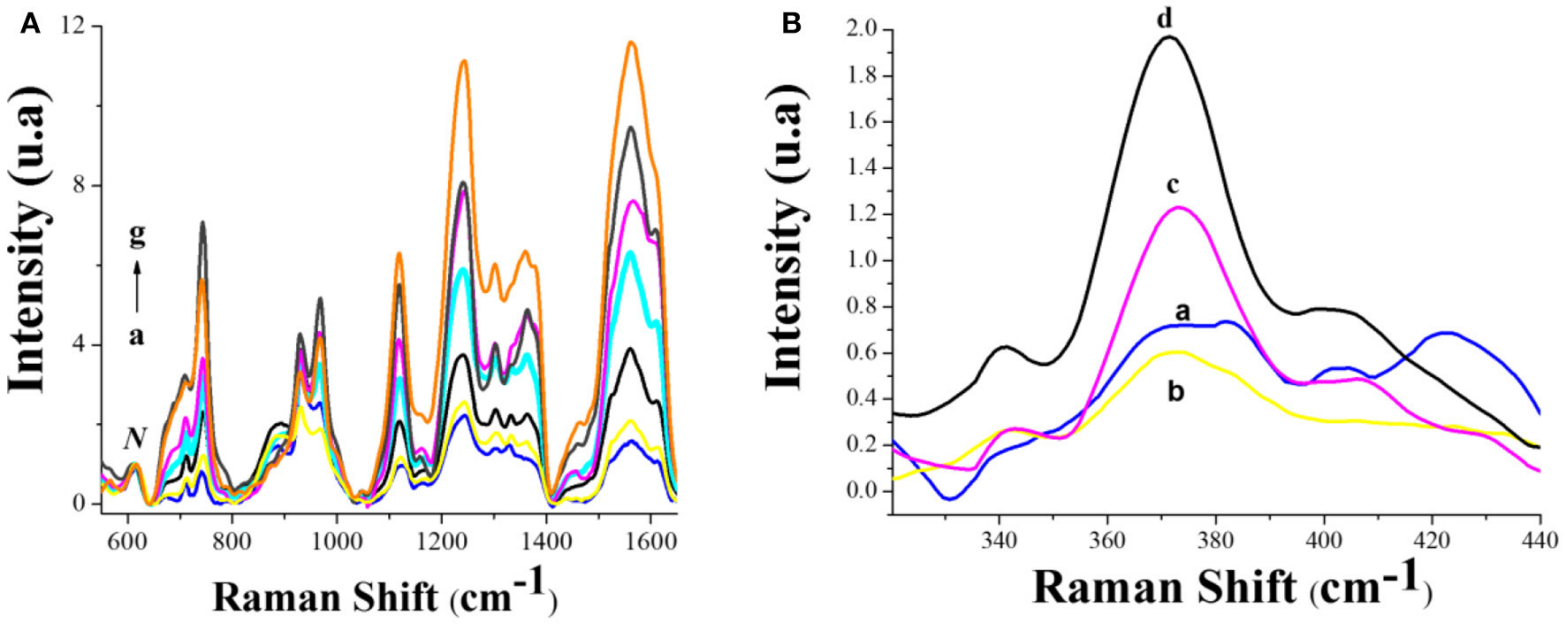
**(A)** SERS spectra from 540 to 1,680 cm^−1^ of hemin-glyphosate incubation mixtures containing different concentrations of GLY: (a) 0, (b) 0.1 nM, (c) 1 nM, (d)10 nM, (e) 100 nM, (f) 1 μM, (g) 10 μM. **(B)** SERS spectra from 500 to 1,700 cm^−1^ of the incubated mixtures of hemin-glyphosate solutions containing different concentrations of GLY: (a) 0, (b) 0.1 nM, (c) 100 nM, (d) 1 μM. *N:* band used for normalization.

The SERS spectra of hemin-glyphosate incubation mixtures containing different concentrations of GLY are reported in Figure 6A. All bands of hemin grow as a function of the glyphosate concentration in solution. It is important to highlight the importance of the incubation time to obtain reproducible Raman signal intensity in the assays since, without 48 h of incubation, the acquisition of a distinguishable trend in the Raman intensity is not possible (see Supplementary Figure 3 in supporting information). In order to understand the origin of the SERS effect that allows the continuous increase of practically all the bands of hemin as a function of glyphosate concentration in the incubated solutions, an in-depth analysis of the SERS experiments was conducted.

From Figure 6A and Table 1, the Eu infrared active mode is indicative of the asymmetry in the vinyl substituents of hemin on the silver nanoparticles (Choi et al., 1982). Moreover, the domain of modes attributable to the vinyl groups [δ(CβCaCb)_4−vinyl_, δ(CβCaCb)_2−vinyl_, ν(Cb-Cα (vinyl) + δ(Cb-R), δ(CaH=), δ(=CbH_2_), ν (Ca=Cb)], implies that hemin is adsorbed on silver through their vinyl substituents in the presence and absence of glyphosate. This can be confirmed with the Raman spectra of the same samples, on the silicon wafer without silver nanoparticles (resonance Raman (rR) experiments), where only a single band of vinyl at 1,615 cm^**–**1^ appears (Supplementary Figure 4 of supporting information).

The band at around 1,614 cm^−1^ contains contributions from the two vinyl side chains of hemin that converge at the same wavenumber value due to the coplanar orientation of both groups with the porphyrin plane (Milazzo et al., 2020). On SERS substrates (see Figure 6A), the wavenumber value of the 1,614 cm^−1^ band undergoes a gradual down-shift when glyphosate concentration is increased in the hemin solution, reaching a value of 1,609 cm^−1^ at the highest tested concentration (1 × 10^−5^ M). However, in the rR spectrum of hemin, the frequency value of this mode remains unchanged in the presence of glyphosate (Supplementary Figure 4). This result suggests a gradual change in the planar orientation of the vinyl groups of hemin due to its binding onto the silver substrate, presenting a major conjugation between the vinyl substituent and the porphyrin macrocycle at the higher concentration of glyphosate (higher degree of trans configuration) (Marzocchi and Smulevich, 2003). Although the resonance response to an increased conjugation should be accompanied by a decrease in the intensity and a down-shift of the wavelength value of the ν2 mode (Rwere et al., 2008), the presence of glyphosate makes this mode to show an increase in intensity without a change in position due to an increase in the proximity of the vinyl groups to the surface of silver (electromagnetic enhanced mechanism).

The evaluation of the high wavenumber range in Figure 6A includes a resurgence of the band *v*4, which becomes well-resolved at the highest glyphosate concentration (1 × 10^−5^ M). The *v*4 mode is associated with the C-N stretch vibrations of the pyrrole subunits and is considered the oxidation-state marker band. The occurrence of *v*4 mode at 1,366 cm^**–**1^ is consistent with the Fe atom in its Fe(II) oxidation state (Wood et al., 2001). The position of *v*15 at 744 cm^**–**1^ corroborates the presence of reduced hemin on the surface of the Si-AgNPs substrates (Zheng et al., 2003).

At low wavenumber values (Figure 6B), the presence of glyphosate provokes the attenuation of intensity in the band at 421 cm^**–**1^ assigned to the out-of-plane bending motion of the vinyl group attached to the pyrrole II group (Rwere et al., 2008). This phenomenon is associated with an increase in its degree of planarity over the surface. As shown in the inset of Supplementary Figure 4, this band does not change in intensity and position in the rR spectra in the presence of glyphosate, suggesting that the change observed on the SERS substrate comes from re-orientation of the 2 vinyl group of the hemin molecule over the surface of the silver substrate. Figure 6B shows an increase of the band at 372 cm^**–**1^ without a change in its position when glyphosate is present in the hemin solutions. The presence of propionate modes under such conditions that are not observed in the insert of Supplementary Figure 4 implies an increase in the nearness of the propionate group to the silver surface. This is only conceived when considering the cofacial π-π dimmer of hemin, where although there is no overlap between the porphyrin nuclei of the two FP-Fe(III) units, an overlap between the vinyl group of one unit and the porphyrin group of a second unit is present (Klonis et al., 2010). Thus, an approach of the vinyl groups of the hemin dimmers to the Ag surface also implies the proximity of the propionate group.

On the other hand, as shown in Figure 6A and Table 1, the enhanced modes of hemin in the presence of glyphosate at an excitation of 780 nm include the A1g and B1g type modes. At this wavelength in resonance Raman spectroscopy experiments on hemin solutions, the A1g and B1g type modes increase in intensity, compared with other excitation wavelengths (Franzen et al., 2002). Therefore, the observed increase in SERS, may come from a resonance Raman phenomenon: a modification in the electronic distribution of hemin as a result of a change in its heme-iron valence, corroborating that these modes arise from a charge transfer (CT) transition of heme by a vibronic coupling mechanism (Wood et al., 2004).

**Figure 7:**
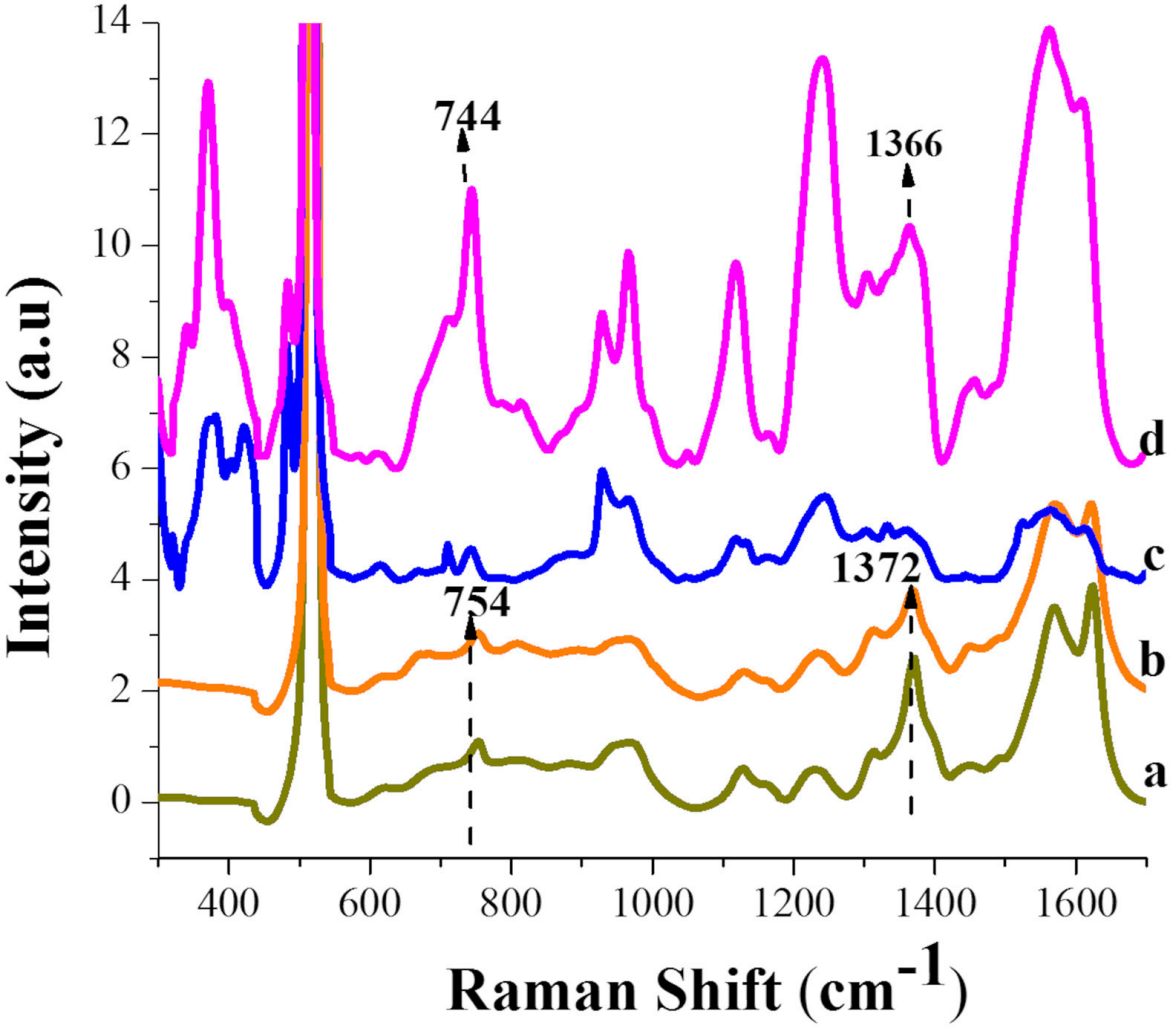
SERS spectra acquired at 532 nm of excitation for: (a) 25 μM of hemin–borate solution, (b) mixture of hemin-borate and 1 μM glyphosate solutions. SERS spectra acquired at 780 nm of excitation for: (c) the same solution as in (a), (d) the same solution as in (b). Range: 540–1680 nm; incubation time: 48 h.

To contrast the results obtained at 780 nm, SERS measurements of the same solutions were recorded using excitation with a laser of 532 nm, where an electronic transition (Q band) and a LSPR absorption signal in the Si-AgNPs substrates also occur. Therefore, in the same way as in the case of excitation at 780 nm, electromagnetic and chemical contributions are expected for the obtained SERS response. Figure 7 shows the SERS spectra obtained at 532 nm of excitation wavelength for hemin borate solution before (curve a) and after (curve b) 48 h of incubation with 10 mM glyphosate solutions. For comparison, the respective SERS spectra obtained with excitation at 780 nm are added (curves c and d).

At 532 nm, the domain of modes attributable to hemin vinylic groups in the absence and presence of glyphosate are observed. However, the position of the C=C mode at 1,620 cm^−1^ and that of the low-frequency modes associated with the bending modes of the vinyl (from 320 to 456 cm^−1^) do not show changes in the presence of glyphosate. This behavior allows inferring that hemin is adsorbed through its vinyl groups, but does not undergo a planar orientation change on silver substrates. The *v*4 mode at 1,372 cm^−1^ indicates the ferric state, Fe(III) (DeVito and Asher, 1989). This information is confirmed by the presence of the ν15 mode at 754 cm^−1^, as considered by Zheng et al. (2003). Thus, from the observed at both 780 and 532 nm excitations, a change in the conformation of the vinyl groups on silver can provoke the affectation of the heme iron redox potential, as it has been inferred in the literature (Chen et al., 2004). On the other hand, it is corroborated that the ν15 mode is also sensitive to the redox state of the heme iron.

### Glyphosate Quantification

**Figure 8:**
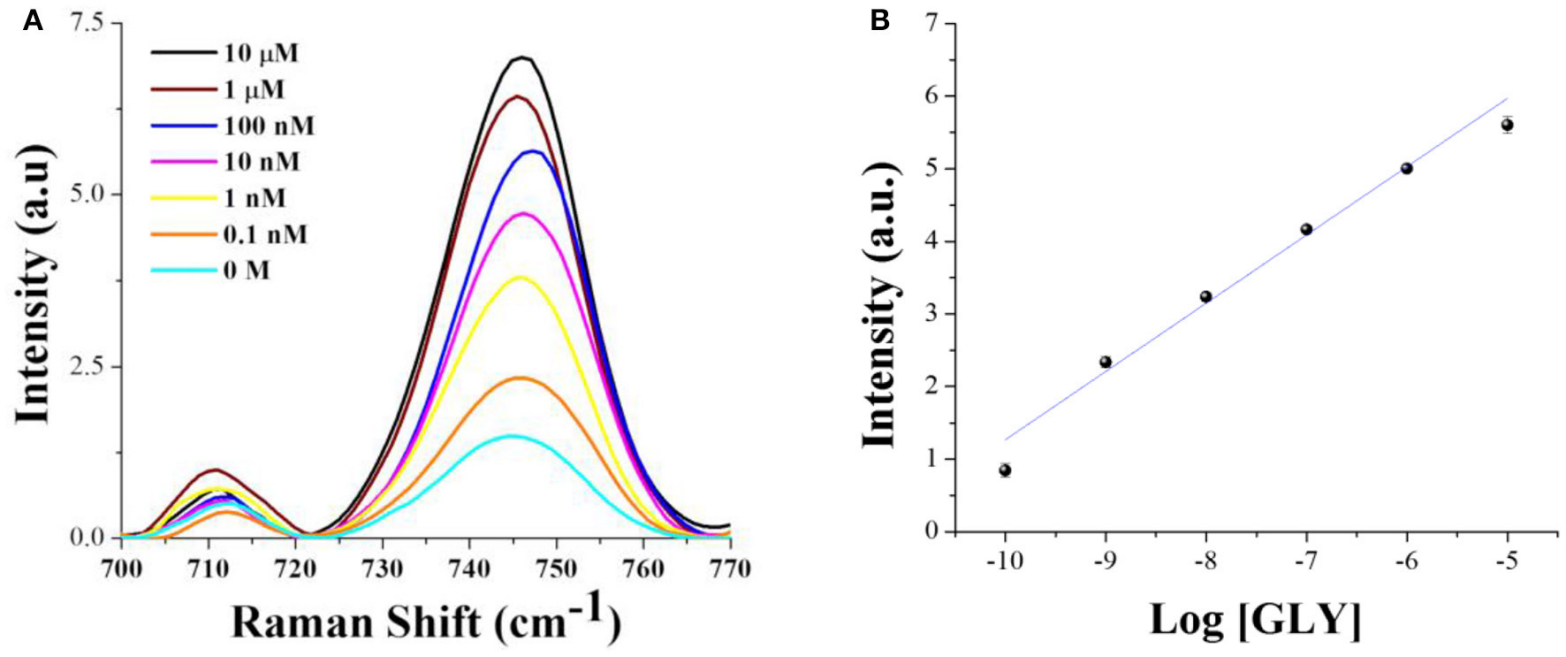
**(A)** SERS spectra at 780 nm excitation wavelength for different glyphosate concentrations in 25 μM of hemin-borate solutions after 48 h of incubation. **(B)** Calibration curve.

Indirect quantification of glyphosate by SERS at 780 nm excitation wavelength was achieved by monitoring the pyridine ring breathing mode of hemin at 745 cm^−1^ with the contribution of the negatively charged borate ion ${\left(\text{B}(\text{OH}\right)}_{4}^{-}$), which increases with the herbicide concentration. It is important to highlight that in the absence of hemin at the same experimental conditions, this band does not show a clear intensity tendency to allow quantification (Supplementary Figure 5). Figure 8A corresponds to the SERS spectra of hemin solutions with different glyphosate concentrations after 48 h of incubation. All the spectra were normalized against the band at 715 cm^−1^, and the intensity values were settled to zero at the foot of the band (720 cm^−1^). Figure 8B shows the calibration curve constructed by plotting the intensity of the bands at 745 cm^−1^ vs. Log[GLY]. The intensity values were corrected by subtracting the intensity of the curve obtained in the absence of glyphosate.

A determination coefficient *R*^2^ of 0.9801 was obtained from the fitted curve, with excellent experimental repeatability as observed from the error bars that comprise the standard deviations from three independent measurements. The equation describing this relationship is:

Intensity = 0.9403 Log[GLY] + 10.6718

The linear range is from 1 × 10^−10^ to 1 × 10^−5^ M. The limit of detection (LOD) was 9.59 × 10^−12^ M (obtained from 3 standard deviations from the average of the blank sample). This value is lower than that found with the Abraxis method (8.87 × 10^−8^ M), which is considered one of the most flexible and versatile enzyme-linked immunosorbent assay (ELISA) to detect glyphosate, and the accuracy is compared to standard liquid chromatography and tandem mass spectrometry methods (Berg et al., 2018). Table 2 shows a comparison of the LOD obtained with different methodologies, including SERS. As it can be noted, the LOD in this work is three and two orders of magnitude higher than in our previously published electrochemical method and the one reported by Cao et al. (2019), respectively, but lower than the other shown methods. Also, the limit of quantification (LOQ) attained in this work was 5.69 × 10^−11^ M.

**Table 2:** LOD values for glyphosate detection from different methodologies.

**Technique**	**LOD (mol L^**−1**^)**	**System/condition**	**References**
Electrochemistry	1.4 × 10^−13^	ITO electrode modified with copper and benzene-1,3,5-tricarboxylic acid	Cao et al., 2019
Electrochemistry	7.92 × 10^−15^	Glassy carbon modified with multiwalled carbon nanotubes and horse-radish peroxidase	Cahuantzi-Muñoz et al., 2019
UV-Vis spectroscopy	5.32 × 10^−6^	Citrate coated silver nanoparticles (colloidal)	De Góes et al., 2017
SERS	1.89 × 10^−5^		
Sequential-injection reversed-phase chromatography	3 × 10^−8^	Pre-column derivatization	Oliveira Pereira et al., 2019
SERS	1.43 × 10^−8^	GLY derivatization with ninhydrin/colloidal silver nanoparticles	Xu et al., 2018
SERS	9.59 × 10^−12^ M	Si-AgNPs substrate/use of hemin as a reporter molecule	This work

### Glyphosate Quantification in Honey

**Figure 9:**
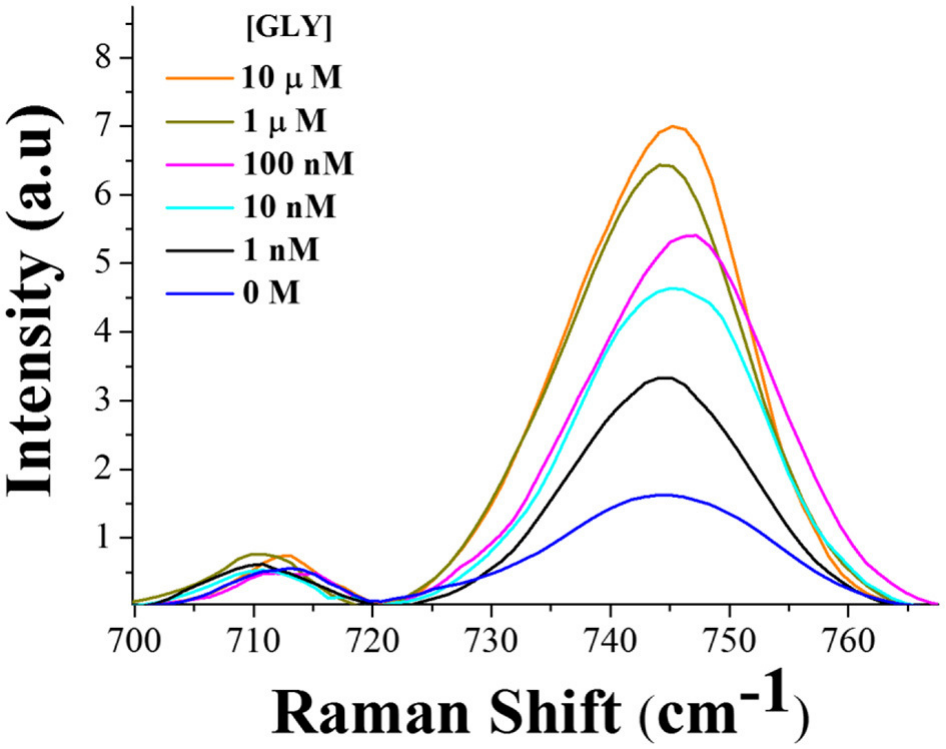
SERS spectra taken at the excitation wavelength of 780 nm for the mixture of hemine 25 μM + borate solution 0.25 M, after 48 h of incubation with different concentrations of glyphosate (0–10 μM) spiked in honey samples.

As an example of the applicability of the quantification proposal, organic honey samples collected from a local market were spiked with different concentrations of glyphosate (1 × 10^−9^, 1 × 10^−8^, 1 × 10^−7^, 1 × 10^−6^, and 1 × 10^−5^ M). Before the hemin incubation step (48 h), which precedes SERS measurements, the pH of honey samples dissolved in borate solution was adjusted to 9.2 with NaOH in order to obtain a homogeneous mixture with hemin. The accuracy and precision of the method were tested via recovery experiments. Figure 9 shows the SERS response of the incubated solutions of honey/hemin in the absence and the presence of different concentrations of glyphosate.

From Figure 9, it is clear that no Raman signal comes from honey or sodium hydroxide in the range from 700 to 770 cm^−1^. Quantification of glyphosate was conducted using the band at 715 cm^−1^ for normalization, and the band at 745 cm^−1^ was employed as the marker band. Table 3 shows the recovery values that are related to accuracy. Recovery values were between 92 and 135% for concentrations from 1 × 10^−9^ to 1 × 10^−6^ M, which is indicative of the absence of matrix effects. A lower recovery (40%) was obtained for the higher concentration surveyed (1 × 10^−5^ M). This fact is not surprising since it is known that SERS measurements can have deviations at high concentrations (Sackmann and Materny, 2006).

**Table 3:** Accuracy and precision obtained with the SERS sensing system.

**Added [GLY] (mol L^**−1**^)**	**Calculated [GLY] (mol L^**−1**^)**	**% Recovery**
1 × 10^−9^	0.866 × 10^−9^	135.20
1 × 10^−8^	0.958 × 10^−8^	122.69
1 × 10^−7^	1.138 × 10^−7^	118.96
1 × 10^−6^	1.061 × 10^−6^	92.29
1 × 10^−5^	0.526 × 10^−5^	40.13

The maximum residue limit (MRL) for glyphosate in honey is established at 50 μg kg^−1^ by the European Union (E. U., 2013). In this work, the linear range is 0.116–1,165 μg kg^−1^ for honey samples, and the EU MRL value is within this interval. The limit of quantification (LOQ) obtained from 10 times the standard deviation of the blank sample average was 11.6 ng kg^−1^, representing values far below the EU MRL. It is important to mention that the scope of this work is to present a concept of glyphosate sensing based on SERS measurements with the use of a reporter molecule (hemin) that can be promising as an alternative methodology. Further studies will include the influence of possible interferents such as glufosinate, aminomethylphosphonic acid (AMPA), and other herbicides.

## Conclusions

In this work, we report a SERS approach for indirect quantification using a reporter molecule (RM) under a previous incubation process with the analyte, a method that has not been reported previously. The key concept is based on implementing an incubation step that allows a binding equilibrium process between the RM and the analyte, since we demonstrated that this step may influence the Raman signal reproducibility, which is an important aspect pursued in this research area. Additionally, because the incubation process is not conducted directly on the SERS substrate, oxidation or dissolution of metallic nanoparticles are prevented. Using this proposal, the quantification of glyphosate at 780 nm of laser excitation wavelength was possible by following the changes in a band that belongs to the hemin used as the RM, resulting in a LOD value as low as 1 × 10^−13^ M. It was also successfully tested in real honey samples without the interference of the sample matrix. On the other hand, it was found that in the presence or absence of glyphosate, hemin is the adsorbed species on SERS substrates through its vinyl groups and undergoes the reduction of its prosthetic group. On the other hand, comparing the results with those using 532 nm, it was corroborated that the position of the ν15 mode of hemin can be used together with the well-known oxidation-state marker band (*v*4 mode) to determine the redox state of heme iron. Finally, the method opens the possibility of exploring the other RM-analyte systems where the binding equilibrium occurs during the incubation process.

## Data Availability Statement

The original contributions presented in the study are included in the article/Supplementary Material, further inquiries can be directed to the corresponding authors.

## Author Contributions

KL-C: conducted SERS experiments. LO-F: conducted UV-Vis experiments and helped in spectra interpretation. EM: conducted AFM measurements and supported with the data treatment and interpretation. EQ-G: elaborated SERS substrates, made reflectance spectroscopy, treated, and discussed data. MG-F: interpreted and discussed results, planned experiments, wrote, and submitted the article. AM-A: interpreted and discussed results, planned experiments, and wrote the article. All authors contributed to the article and approved the submitted version.

## Conflict of Interest

The authors declare that the research was conducted in the absence of any commercial or financial relationships that could be construed as a potential conflict of interest.
