# Supplementary material for: Indirect Quantification of Glyphosate by SERS Using an Incubation Process With Hemin as the Reporter Molecule: A Contribution to Signal Amplification Mechanism
Source: Front Chem. 2020 Dec 18;8:612076. doi: 10.3389/fchem.2020.612076 (PMC7775572; doi:10.3389/fchem.2020.612076)
Supplement: Supplementary file 1 [file Data_Sheet_1.docx]

**Supporting information**

Indirect quantification of glyphosate by SERS using an incubation process with hemin as the reporter molecule: A contribution to signal amplification mechanism

**Karen A. López-Castaños^1^, Luis A. Ortiz-Frade^2^, Erika Méndez^3^, Enrique Quiroga-González^4^, Miguel A. González-Fuentes^3*^, Alia Méndez-Albores^1*^**

^1^Centro de Química-ICUAP, Benemérita Universidad Autónoma de Puebla, Puebla, Mexico

^2^Centro de Investigación y Desarrollo Tecnológico en Electroquímica (CIDETEQ), Pedro Escobedo, Querétaro, Mexico

^3^Facultad de Ciencias Químicas, Benemérita Universidad Autónoma de Puebla, Puebla, Mexico

^4^Institute of Physics, Benemérita Universidad Autónoma de Puebla, Puebla, Mexico

***Correspondence:**

Alia Méndez-Albores

[alia.mendez@correo.buap.mx](mailto:alia.mendez@correo.buap.mx)

Miguel A. González-Fuentes

[miguel.gonzalezfuentes@correo.buap.mx](mailto:miguel.gonzalezfuentes@correo.buap.mx)

**Figure S1.** Absorbance spectra of freshly prepared hemin solutions at different concentrations in 0.25 M borate solution. Soret (B), Q (I, II) and CT (III, IV, V) bands are indicated in the graph.

**Figure S2.** Raman spectra at 780 nm excitation wavelength of different systems from 320 to 1671 cm^−1^

**Figure S3.** Dependence of Raman intensity as a function of incubation time, for the hemin-glyphosate mixture. The concentration of GLY in the mixture corresponds to the highest tested (10 μM), with which the mixture undergoes the maximum standard deviations of Raman intensity values. Additionally, from this graph, 48 h was selected as the incubation time required to obtain the best repeatability, since it was also true for the rest of GLY concentrations.

**Figure S4.** Raman spectra using the "*drop technique*" on silicon wafer for the systems: 25 μM hemin in 0.25 M borate solution (curve a), hemin-glyphosate mixture containing 1 μM of glyphosate (curve b). *v*12 mode corresponds to the in-plane skeletal frequency of the B_lg_ symmetry, assigned to the n(Pyr. half-ring)sym mode (Spiro et al., 1990). Insert: Same spectra in the range 320-440 cm^-1^.

**Figure S5.** SERS spectra at 780 nm of excitation wavelength for different concentrations of glyphosate in 0.25 M borate solution after 48 h of incubation.

**Reference**

Spiro, Thomas G, Roman S Czernuszewicz, and Xiao-Yuan Li. "Metalloporphyrin Structure and Dynamics from Resonance Raman Spectroscopy." Coordination chemistry reviews 100 (1990): 541-71.
